# Safety and efficacy of radium-223 dichloride in Japanese patients with castration-resistant prostate cancer and bone metastases

**DOI:** 10.1007/s10147-017-1130-1

**Published:** 2017-05-06

**Authors:** Hiroji Uemura, Hirotsugu Uemura, Nobuaki Matsubara, Seigo Kinuya, Makoto Hosono, Yoko Yajima, Toshihiko Doi

**Affiliations:** 1 Department of Urology and Renal Transplantation, Yokohama City University Medical Center, 4-57 Urafune-cho, Minami-ku, Yokohama, Japan; 2 Department of Urology, Kindai University Faculty of Medicine, Osaka, Japan; 3 Department of Breast and Medical Oncology, National Cancer Center Hospital East, Kashiwa, Japan; 4 The Japanese Society of Nuclear Medicine, Tokyo, Japan; 5 Clinical Development Specialty Medicine, Product Development, Bayer Yakuhin, Ltd., Tokyo, Japan; 6 Department of Gastroenterology and Gastrointestinal Oncology, National Cancer Center Hospital East, Kashiwa, Japan

**Keywords:** Castration-resistant prostate cancer, Efficacy, Japanese patients, Radium-223 dichloride, Safety

## Abstract

**Background:**

Radiation therapy with radium-223 dichloride improves overall survival, reduces symptomatic skeletal events in Caucasian patients with castration-resistant prostate cancer (CRPC) and bone metastases, and is well tolerated. We report here the results of the first efficacy and safety study of radium-223 dichloride in a Japanese population.

**Methods:**

In this open-label, uncontrolled, non-randomized, phase I trial, radium-223 dichloride was given to Japanese patients with CRPC and ≥2 bone metastases in 4-week cycles. The patients were divided into three cohorts, with cohort 1 and the expansion cohort receiving injections of radium-223 dichloride [55 kBq/kg body weight (BW)] every 4 weeks (Q4W) for up to six injections, and cohort 2 receiving an initial single radium-223 dichloride injection of 110 kBq/kg BW followed by up to five injections of 55 kBq/kg BW Q4W. Safety was determined via adverse event (AE) reporting, and biochemical bone markers were assessed for treatment efficacy.

**Results:**

In total 19 patients received at least one dose of radium-223 dichloride and 18 patients experienced at least one treatment-emergent AE (TEAE) of which the most common were anemia, thrombocytopenia, and lymphocytopenia. Serious AEs were reported in three patients but none were drug-related. In the patients of cohort 1 + expansion cohort (55 kBq/kg BW Q4W treatment; *n* = 16), prostate-specific antigen levels remained stable or slightly increased while the bone alkaline phosphatase (ALP) level significantly decreased. The response rates of bone ALP (≥30 and ≥50% reductions) were 81.8 and 36.4% at week 12, and 81.3 and 50.0% at the end of treatment.

**Conclusions:**

Radium-223 dichloride was well tolerated in these Japanese patients and, at a dose of 55 kBq/kg BW, efficacy on biomarkers was as expected. The outcomes in Japanese patients were consistent with those reported in other non-Japanese populations.

**Trial registration:**

ClinicalTrials.gov record NCT01565746.

## Introduction

Prostate cancer is the second most common cancer in males worldwide and accounts for 15% of all cancers diagnosed in men. It represents the fifth leading cause of death from cancer in men and 6.6% of total male mortality [1]. Among patients with localized prostate cancer, treatments are effective, and 5-year survival rates are approximately 100%. Nevertheless, those with distant metastases often become resistant to treatment, and the 5-year survival rate is considerably lower at 31% among this patient population [2].

 The standard therapy for patients with advanced prostate cancer is androgen deprivation therapy, which includes medical or surgical castration [2, 3]. The disease is defined to be castrate-resistant prostate cancer (CRPC) if it progresses, either biochemically or radiologically, despite serum testosterone levels of <1.7 nmol/L. Within 5 years of follow-up, 10–20% of patients with prostate cancer develop CRPC [4]. In patients with CRPC, the most frequent site of metastases is bone, and comorbidities or skeletal-related events (SREs) caused by bone metastases are associated with deterioration of the quality of life and an increased risk of death [5]. Therefore, the treatment goal for patients with CRPC and bone metastases should be maintaining quality of life, preventing SREs, and improving survival [6].

While a number of different treatment approaches are available for the management of metastatic CRPC, including abiraterone, enzalutamide, docetaxel, cabazitaxel, and sipuleucel-T, the effects of these drugs on bone metastases has not been thoroughly investigated [7]. The active form of radium-223 dichloride is an α-emitting radionuclide and a calcium mimetic that forms complexes with the bone mineral hydroxyapatite at areas of high bone turnover, a typical characteristic of bone metastases. Once at the site of bone metastases, radium-223 dichloride emits α particles and induces breaks in double-stranded DNA, killing tumor cells in a targeted fashion [8, 9]. Radium-223 dichloride was approved by the U.S. Food and Drug Administration in 2013 for the treatment of patients with CRPC and symptomatic bone metastases with no known visceral metastases [8].

Clinical trials in Caucasian patients with CRPC and bone metastases have shown that radium-223 dichloride is well tolerated, improves overall survival, and reduces symptomatic skeletal events (SSEs) [10–12]. The aim of this phase I study was to investigate the pharmacokinetics, dosimetry, safety, and efficacy of radium-223 dichloride in Japanese patients with CRPC and bone metastases. The pharmacokinetic results of this study have been published [13]; we report here the safety and efficacy (biomarker) outcomes of the study.

## Patients and methods

### Selection of patients

#### Inclusion criteria

The study population included male patients aged ≥20 years with histologically-confirmed adenocarcinoma of the prostate, with ≥2 bone metastases confirmed by scintigraphic imaging within the 4 weeks preceding the start of radium-223 dichloride treatment, and who had failed initial hormonal therapy. Other inclusion criteria were: (1) castrate levels of testosterone of <50 ng/dL (1.7 nmol/L) and continued treatment to maintain castrate levels of testosterone; (2) progressive castration-resistant metastatic disease, defined as at least one of the following: new osseous lesions observed via radionuclide bone scan, a ≥20% increase in the sum of the longest diameter of target lesions, or ≥3 rising prostate specific antigen (PSA) values from baseline; (3) Eastern Cooperative Oncology Group performance status 0–2; (4) alkaline phosphatase (ALP) level greater than the upper institutional limit of normal range.

#### Exclusion criteria

Patients were excluded from the study if they had (1) received an investigational drug in the 4 weeks immediately preceding the start of radium-223 dichloride treatment, or were scheduled to receive one during the treatment or 8 weeks after study drug administration; (2) received chemo-, immuno-, or radiotherapy within the last 4 weeks prior to entry in the study, or had not recovered from acute adverse events (AEs) as a result of such therapy; (3) started or stopped systemic steroids within 1 week prior to study drug administration, or were expected to change systemic steroids; (4) had a history of gastrointestinal bleeding or ulcer within 3 months prior to study entry; (5) had small cell carcinoma; predominant visceral metastases (≥3 lung or liver lesions) or symptomatic lymphadenopathy which was characterized by scrotal or pedal edema.

Written informed consent was obtained from all the patients or their legally authorized representatives prior to the study.

### Study design

This study was an open-label, uncontrolled, non-randomized, multicenter phase I trial (Trial registration: ClinicalTrials.gov number NCT01565746) conducted at three study centers in Japan (National Cancer Center Hospital East, Yokohama City University Hospital, and Kinki University Hospital).

All patients received a single intravenous bolus of radium-223 dichloride. A single 50 kBq/kg body weight (BW) dose (equivalent to 55 kBq/kg BW after implementation of the National Institute of Standards and Technology (NIST) update [14]; hereafter described as 55 kBq/kg) was given to patients in cohort 1, and if the incidence of critical toxicity was lower than 33%, a single dose of 100 kBq/kg BW (equivalent to 110 kBq/kg BW after the NIST update, and hereafter described as 110 kBq/kg) was given to cohort 2 (cycle 1). Cycle 2 and subsequent 4-week cycles (at a dose of 55 kBq/kg) continued for up to five additional doses for cohort 1 and up to four additional doses for cohort 2. Patients were allowed to receive the next dose only if they did not have definitive progressive disease and did not show critical toxicity.

Additional patients were enrolled in the expansion cohort provided the safety of radium-223 dichloride was confirmed in cohort 1. The patients in cohorts 1 and 2 were hospitalized for the first 28 days, while those in the expansion cohort were hospitalized for the first 8 days for safety observations. All patients were followed up at 4, 8 and 12 weeks after the last treatment, plus every 6 months after the last treatment for up to 36 months after the first treatment.

The study was conducted according to four internal manuals outlining a standard protocol for the proper use of radium-223 dichloride, describing (1) the safe and efficient use of medical radiation [15], (2) proper use of radionuclide therapy in clinical trials 1, (3) protection from medical radiation [16], and (4) quantifying shielding and radiation exposure in the atmosphere, exhaust air and exhaust fluid 2.

 All study protocols were approved by the Institutional Review Boards of the National Cancer Center Hospital East, Yokohama City University Hospital and Kinki University Hospital before commencing the study. In addition to all local legal and regulatory requirements, the study was conducted in accordance with the Declaration of Helsinki and the International Conference on Harmonization guideline E6: Good Clinical Practice.

### Study outcomes

The primary study endpoint/outcome was safety (AEs), while the secondary endpoints included treatment efficacy (determined via biochemical bone markers).

#### Safety assessments

All AEs that occurred in the patients during the study treatment and within 12 weeks after the last dose were recorded. Any causal relationship between the given treatment and observed AEs was assessed. All AEs were coded by MedDRA Version 16.1 (https://www.meddra.org/sites/default/files/.../intguide_16_1_english.pdf) and graded according to the National Cancer Institute Common Terminology Criteria for Adverse Events (version 4.0) (https://evs.nci.nih.gov/ftp1/CTCAE/CTCAE_4.03_2010-06-14_QuickReference_5x7.pdf). The critical toxicities were defined as (1) grade 3 or higher non-hematologic toxicity or (2) hematologic toxicity, such as grade 3 neutropenia with fever or grade 4 neutropenia that failed to recover to grade 2 or less after treatment with granulocyte-colony stimulating factor within 2 weeks or (3) grade 4 thrombocytopenia.

A serious AE (SAE) was one that was life-threatening, required inpatient hospitalization or prolongation of existing hospitalization, or resulted in persistent or significant disability/incapacity, a congenital anomaly, serious event, or death.

Treatment-emergent AEs (TEAEs) were defined as all events occurring or worsening after the first injection and within 30 days after the last injection of radium-223 dichloride.

Post-treatment follow-up AEs were recorded for 30 days after the last dose up to 12 weeks after the last dose. AEs which occurred and were considered to be related to treatment with radium-223 dichloride were reported every 6 months after the last dose for up to 36 months after the first dose.

#### Efficacy assessments

Levels of PSA and bone markers, including serum total ALP, serum bone ALP, procollagen 1 *N*-terminal propeptide (P1NP), C-terminal crosslinked telopeptide of type I collagen (CTX-1), and carboxyterminal telopeptide of type I collagen (ICTP) were used for the efficacy assessment. All markers were measured at screening, at baseline before injection, on day 15 in cycle 1, on day 1 in cycle 2 and subsequent cycles, at the end of treatment (EOT), and at 4, 8 and 12 weeks after the last treatment or the end of follow-up.

### Statistical analyses

Statistical analyses for the study were performed using the Statistical Analysis System (SAS; SAS Institute Inc., Raleigh, NC). The safety analysis included all patients who received at least one dose of study medication, while the efficacy analysis included all patients who received at least one dose and who had post-baseline efficacy data available. Demographic and other baseline characteristics were summarized using descriptive statistics. The PSA values, changes from baseline, and percentage changes from baseline were summarized by visit. Response rates (≥30% reduction and ≥50% reduction) were estimated at 12 weeks and at the EOT for PSA and bone markers.

## Results

### Patient disposition and baseline characteristics

A total of 19 patients were enrolled in the study. All received at least one dose of radium-223 dichloride and were included in the safety and efficacy analysis set (three in cohort 1, three in cohort 2, and 13 in the expansion cohort). Demographic and baseline characteristics and any prior treatment received by the study patients are shown in Table 1.

**Table 1:** Demographics, baseline characteristics, and prior treatments

Patient characteristics	Cohort 1 (*n* = 3)	Cohort 2 (*n* = 3)	Expansion cohort (*n* = 13)	Cohort 1 + expansion cohort (*n* = 16)	Total (*n* = 19)
Demographic characteristics, mean ± SD					
Age (years)	73.3 ± 6.7	71.7 ± 5.9	71.3 ± 4.7	71.7 ± 4.9	71.7 ± 4.9
Weight (kg)	67.7 ± 4.2	60.1 ± 3.1	62.3 ± 7.8	63.3 ± 7.5	62.8 ± 7.0
Height (cm)	162.5 ± 4.8	165.8 ± 4.1	163.2 ± 4.2	163.1 ± 4.2	163.5 ± 4.2
Body mass index (kg/m^2^)	25.6 ± 0.5	21.9 ± 1.1	23.4 ± 3.8	23.8 ± 3.2	23.5 ± 3.0
ECOG performance status at baseline, *n* (%)					
0	3 (100.0)	3 (100.0)	11 (84.6)	14 (87.5)	17 (89.5)
1	0	0	2 (15.4)	2 (12.5)	2 (10.5)
Prior anticancer therapy/therapeutic procedures, *n* (%)					
Prior therapeutic procedure^a^	1 (33.3)	1 (33.3)	2 (15.4)	3 (18.8)	4 (21.1)
Prior diagnostic procedure^b^	3 (100.0)	3 (100.0)	13 (100.0)	16 (100.0)	19 (100.0)
Prior systemic anti-cancer therapy	3 (100.0)	3 (100.0)	13 (100.0)	16 (100.0)	19 (100.0)
Prior radiotherapy	0	1 (33.3)	3 (23.1)	3 (18.8)	4 (21.1)
Prior local anti-cancer therapy^c^	0	0	0	0	0
Baseline of tumor markers, mean ± SD					
PSA (ng/mL)	42.8 ± 25.1	669.6 ± 737.5	379.7 ± 505.5	316.5 ± 472.2	372.3 ± 496.2
ALP (U/L)	198.0 ± 52.8	1354.0 ± 1697.8	1024.1 ± 1015.6	869.2 ± 967.6	945.7 ± 1049.0

*ALP* Alkaline phosphatase, *ECOG* Eastern Cooperative Oncology Group, *PSA* prostate specific antigen,* SD* standard deviation

^a^Prior therapeutic procedure includes orchiectomy and/or prostatectomy

^b^Prior diagnostic procedure includes biopsy and/or prostatectomy

^c^Local anticancer therapy includes radiotherapy and surgery

### Treatment exposure

The median duration of radium-223 dichloride treatment ranged from 114 to 142 days in all three cohorts, with patients receiving a median of five or six injections. The median total dose of radium-223 dichloride ranged from 15,736 kBq in the expansion cohort to 22,214 kBq in cohort 1. For the 55 kBq/kg treatment (cohort 1 + expansion cohort, *n* = 16) the median duration of treatment and number of injections was 129 days and 5.5 injections, respectively; the median total dose of radium-223 dichloride that patients received was 18,983 kBq. For the 110 kBq/kg treatment (cohort 2, *n* = 3) the median duration of treatment and number of injections was 114 days and 5.0 injections, respectively; the median total dose of radium-223 dichloride that patients received was 18,778 kBq.

### Safety

Almost all patients (*n* = 18, 94.7%) experienced one or more TEAEs; those TEAEs considered to be drug-related are summarized in Table 2. No grade 4 or grade 5 TEAEs were observed (Table 3). The grade 3 TEAE occurring in the highest proportion of patients was anemia (21.1%, 4/19), while other TEAEs were observed in one patient (5.3%). Three patients died in the post-treatment period in the expansion cohort (23.1%, 3/13), and no deaths were observed in cohort 1 or cohort 2. All deaths were considered to be unrelated to study treatment. SAEs were experienced by three patients in the expansion cohort (23.1%, 3/13) during the treatment period (Table 4). The worst grade of these SAEs was grade 3 (infection, lung infection, bone pain, and prostate cancer), and grade 2 (rectal hemorrhage). No SAEs were related to study treatment.

**Table 2:** List of drug-related treatment-emergent adverse events

TEAEs, *n* (%)	Cohort 1 (*n* = 3)	Cohort 2 (*n* = 3)	Expansion cohort (*n* = 13)	Cohort 1 + expansion cohort (*n* = 16)	Total (*n* = 19)
Drug-related TEAEs^a^					
Any	1 (33.3)	3 (100.0)	7 (53.8)	8 (50.0)	11 (57.9)
Worst grade, grade 5 (death)	0	0	0	0	0
Worst grade, grade 3 or 4^b^	0	0	2 (15.4)	2 (12.5)	2 (10.5)
Drug-related post treatment follow-up AEs^c^					
Any	0	0	2 (15.4)	2 (12.5)	2 (10.5)
Grade 5 (death)	0	0	0	0	0
Grade 3 or 4^b^	0	0	1 (7.7)	1 (6.3)	1 (5.3)
Long-term toxicity^d^	0	0	0	0	0
All drug-related TEAEs in treatment period, by MedDRA term (and by CTCAE where different)					
Any	1 (33.3)	3 (100.0)	7 (53.8)	8 (50.0)	11 (57.9)
Anemia	1 (33.3)	0	3 (23.1)	4 (25.0)	4 (21.1)
Constipation	0	0	1 (7.7)	1 (6.3)	1 (5.3)
Diarrhea	0	3 (100.0)	0	0	3 (15.8)
Lymphocytopenia (lymphocyte count decreased)	0	0	2 (15.4)	2 (12.5)	2 (10.5)
Thrombocytopenia (platelet count decreased)	1 (33.3)	0	2 (15.4)	3 (18.8)	3 (15.8)
Leukopenia (white blood cells decreased)	0	0	1 (7.7)	1 (6.3)	1 (5.3)
Bone pain	0	0	1 (7.7)	1 (6.3)	1 (5.3)
Dysgeusia	0	0	2 (15.4)	2 (12.5)	2 (10.5)
Rash (rash acneiform)	0	1 (33.3)	0	0	1 (5.3)

*AEs* adverse events, *CTCAE* Common Terminology Criteria for Adverse Events, *MedDRA* medical dictionary for regulatory activities, *TEAEs* treatment-emergent adverse events

^a^TEAEs were defined as all events occurring or worsening after the first injection of study treatment and within 12 weeks after the last injection of study treatment

^b^The worst grade was grade 3; no grade 4 TEAEs were reported

^c^Post-treatment follow-up AEs were defined as AEs considered to be related to the study treatment which occurred between 30 days and 12 weeks after the last treatment or up to the end of the follow-up

^d^Long-term toxicity was defined as AEs considered to be related to the study treatment which occurred between 12 weeks after the last treatment and 36 months after the first treatment

**Table 3:** Grade 3 treatment-emergent adverse events

Grade 3 or grade 4 TEAEs by MedDRA (and by CTCAE where different), *n* (%)	Worst CTCAE grade	Cohort 1 (*n* = 3)	Cohort 2 (*n* = 3)	Expansion cohort (*n* = 13)	Cohort 1 + expansion cohort (*n* = 16)	Total (*n* = 19)
Anemia	Grade 3	0	0	4 (30.8)	4 (25.0)	4 (21.1)
Nausea	Grade 3	0	0	1 (7.7)	1 (6.3)	1 (5.3)
Rectal stenosis	Grade 3	0	0	1 (7.7)	1 (6.3)	1 (5.3)
Infection (infections and infestations—other)	Grade 3	0	0	1 (7.7)	1 (6.3)	1 (5.3)
Lung infection (lung infection)	Grade 3	0	0	1 (7.7)	1 (6.3)	1 (5.3)
Lymphocytopenia (lymphocyte count decreased)	Grade 3	0	0	1 (7.7)	1 (6.3)	1 (5.3)
Leukopenia (white blood cells decreased)	Grade 3	0	0	1 (7.7)	1 (6.3)	1 (5.3)
Decreased appetite (anorexia)	Grade 3	0	0	1 (7.7)	1 (6.3)	1 (5.3)
Inadequate control of diabetes mellitus (glucose intolerance)	Grade 3	0	0	1 (7.7)	1 (6.3)	1 (5.3)
Hypocalcemia	Grade 3	0	0	1 (7.7)	1 (6.3)	1 (5.3)
Hypophosphatemia	Grade 3	0	0	1 (7.7)	1 (6.3)	1 (5.3)
Bone pain	Grade 3	0	0	1 (7.7)	1 (6.3)	1 (5.3)
Cancer pain (tumor pain)	Grade 3	0	0	1 (7.7)	1 (6.3)	1 (5.3)
Prostate cancer (neoplasms benign, malignant and unspecified, including cysts and polyps—other)	Grade 3	0	0	1 (7.7)	1 (6.3)	1 (5.3)
Renal impairment (renal and urinary disorders—other)	Grade 3	0	0	1 (7.7)	1 (6.3)	1 (5.3)

**Table 4:** All treatment-emergent serious adverse events reported during the study

Treatment-emergent SAEs, by MedDRA (and by CTCAE where different), *n* (%)	Cohort 1 (*n* = 3)	Cohort 2 (*n* = 3)	Expansion cohort (*n* = 13)	Cohort 1 + expansion cohort (*n* = 16)	Total (*n* = 19)
Rectal hemorrhage	0	0	1 (7.7)	1 (6.3)	1 (5.3)
Infection (infections and infestations—other)	0	0	1 (7.7)	1 (6.3)	1 (5.3)
Lung infection (lung infection)	0	0	1 (7.7)	1 (6.3)	1 (5.3)
Bone pain	0	0	1 (7.7)	1 (6.3)	1 (5.3)
Prostate cancer (neoplasms benign, malignant and unspecified incl. cysts and polyps—other)	0	0	1 (7.7)	1 (6.3)	1 (5.3)

*SAEs* Serious adverse events

Two patients (10.5%, 2/19) experienced AEs (grade 2 thrombocytopenia and grade 3 gastric hemorrhage, respectively) that led to discontinuation of study treatment in the expansion cohort, but not in cohort 1 or cohort 2. The incidence of serious TEAEs leading to dose interruption and permanent discontinuation of study drug was 15.4% (2/13) and 7.7% (1/13), respectively. One patient from cohort 2 and eight patients from the expansion cohort were withdrawn from the study due to disease progression.

In the expansion cohort, two patients (10.5%, 2/19) reported drug-related post-treatment AEs, including anemia in two patients (grade 2 and 3, respectively), and platelet count decreased in one patient (grade 4). No long-term toxicity was reported in this study.

### Efficacy

**Fig. 1:**
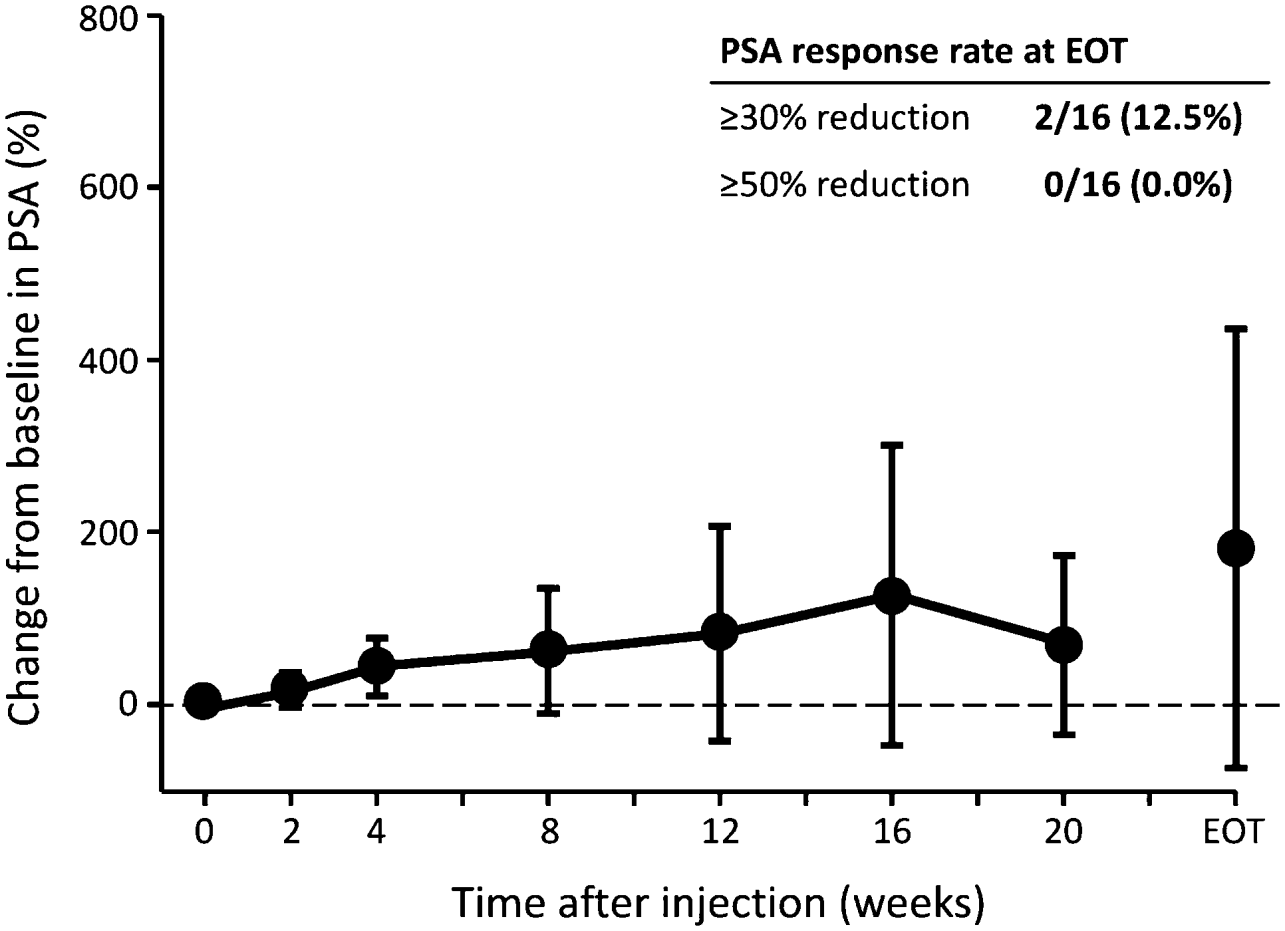
Percentage changes from baseline in prostate-specific antigen (*PSA*) levels after treatment with radium-223 dichloride at 50 kBq/kg (cohort 1 + expansion cohort, *n* = 16).* Filled circles* Mean ± standard deviation (SD). *EOT* End of treatment. PSA response rate was defined as the percentage of patients whose PSA blood level was reduced by ≥30 or ≥50% vs. baseline

In cohort 1 + the expansion cohort, serum PSA levels remained stable or slightly increased after the injection of radium-223 dichloride at week 12 and at EOT (Table 5; Fig. 1).

**Table 5:** Percentage change from baseline in efficacy markers following injections of radium-223 dichloride in cohort 1 + expansion cohort (*n* = 16)

Markers	12 weeks after treatment			End of treatment		
	*n*	Mean ± SD	Range	*n*	Mean ± SD	Range
PSA	11	83.5 ± 124.5	−32.4 to 423.8	16	182.0 ± 254.2	−37.8 to 934.5
Bone markers						
Total ALP	11	−30.4 ± 23.6	−69.1 to 12.1	16	−27.7 ± 25.2	−66.5 to 26.0
Bone ALP	11	−46.2 ± −18.7	−78.0 to −10.5	16	−48.2 ± 17.2	−75.1 to −13.7
P1NP	11	−42.1 ± 25.0	−71.2 to 9.7	16	−29.5 ± 40.0	−81.4 to 45.9
CTX-1	11	−20.8 ± 24.1	−66.7 to 0.0	16	35.9 ± 127.2	−66.7 to 500.0
ICTP	11	14.3 ± 38.3	−12.8 to 116.0	16	69.2 ± 190.4	−24.4 to 763.0

**Fig. 2:**
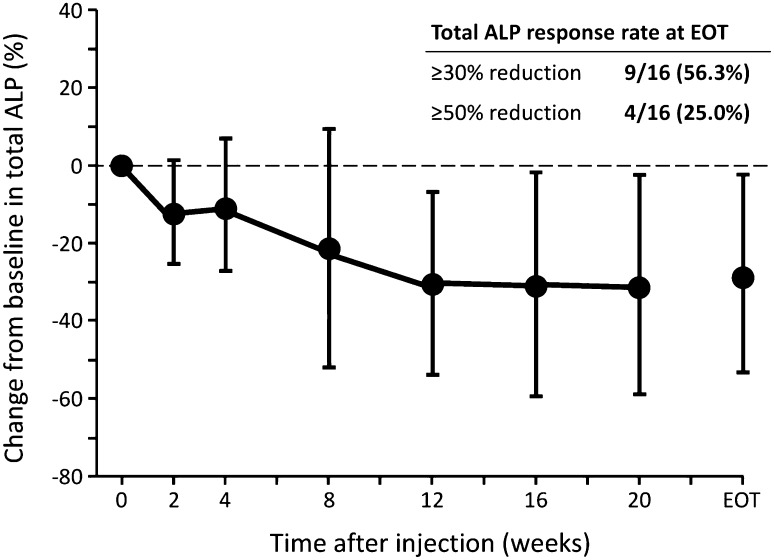
Percentage changes from baseline in total alkaline phosphatase (*ALP*) levels after treatment with radium-223 dichloride at 50 kBq/kg (cohort 1 + expansion cohort, *n* = 16).* Filled circles* Mean ± SD. ALP response rate was defined as the percentage of subjects whose ALP blood level was reduced by ≥30 or ≥50% vs. baseline

**Fig. 3:**
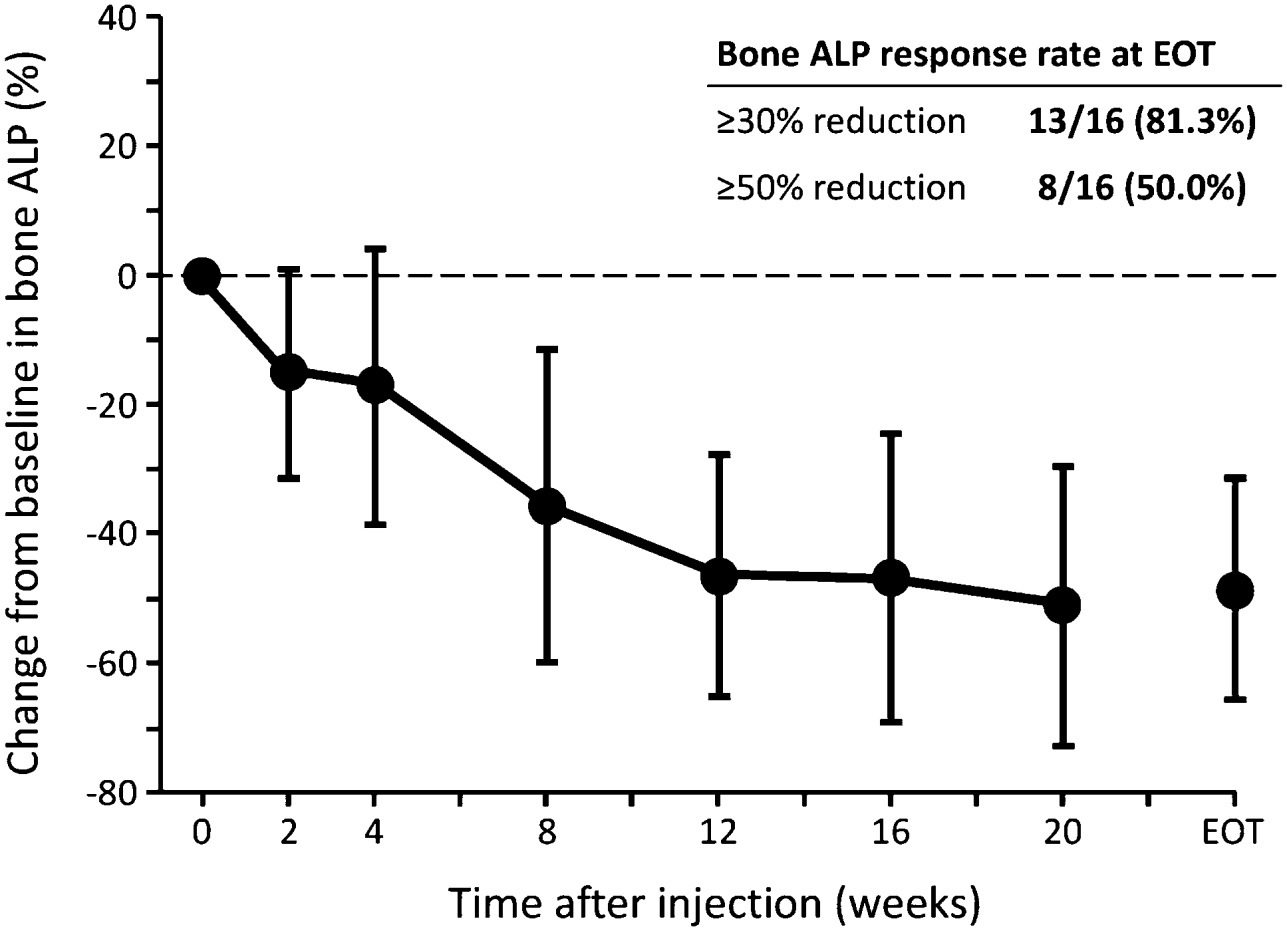
Percentage changes from baseline in bone ALP levels after treatment with radium-223 dichloride at 50 kBq/kg (cohort 1 + expansion cohort, *n* = 16).* Filled circles* mean ± SD. ALP response rate was defined as the percentage of subjects whose ALP blood level was reduced by ≥30 or ≥50% vs. baseline

*CTX-1* C-terminal crosslinked telopeptide of type I collagen, *ICTP* carboxyterminal telopeptide of type I collagen, *P1NP* procollagen 1 N-terminal propeptide,

Total ALP levels in blood decreased from baseline to week 12 and EOT in all the cohorts (Table 5; Fig. 2). The total ALP response rates (≥30 and ≥50% reductions) were 54.5% (6/11) and 9.1% (1/11), respectively, at week 12, and 56.3% (9/16) and 25.0% (4/16), respectively, at EOT. Bone ALP levels also decreased from baseline to week 12 and EOT in all cohorts (Table 5; Fig. 3). Bone ALP response rates (≥30 and ≥50% reductions) were 81.8% (9/11) and 36.4% (4/11), respectively, at week 12, and 81.3% (13/16) and 50.0% (8/16), respectively, at EOT.

The mean percentage change of P1NP from baseline at week 12 and at EOT was −42.1 and −29.5%, respectively. As for the bone resorption markers, the mean percentage change of CTX-I from baseline at week 12 and at EOT was −20.8 and 35.9%, respectively, and that of ICTP was 14.3 and 69.2%, respectively (Table 5).

## Discussion

The safety results of this study show that radium-223 dichloride was well tolerated by the Japanese patients with CRPC and bone metastases who were enrolled in the trial. As such, these results are comparable with those from previous studies in Caucasian patients and confirm the safety results obtained in the early development studies (BC1-05, BC1-08) [17, 18] and the ALSYMPCA study [10]. Of the 19 subjects participating in the study, 18 experienced an AE during the study period, with anemia, diarrhea, and thrombocytopenia being the most frequently observed AEs. The severity of the AEs were grade 1 or 2 in most cases, and no grade 4 or 5 TEAEs were observed. None of the observed treatment-emergent SAEs or AEs that led to discontinuation of study treatment were considered to be drug related. In the ALYSYMPCA study, the overall incidence of AEs in the radium-223 dichloride arm was comparable to or lower than that in the placebo arm [10]. Myelosuppression was rare in patients enrolled in the ALSYMPCA study, with a similar incidence of anemia between patients receiving radium-223 dichloride and those receiving placebo (31% for all grades), and the incidence of thrombocytopenia and neutropenia was 12 and 5%, respectively, in the radium-223 dichloride arm, and 6 and 1%, respectively, in the placebo arm [10]. In a phase II study, radium-223 dichloride improved overall survival, while there were no drug-related AEs or long-term hematological toxicity reported during the 12- to 24-month follow-up period after treatment [12]. In addition, there were no significant differences between the radium-233 dichloride and placebo groups in hematological parameters [12]. Both total and bone ALP are known bone markers that are associated with the diagnosis of bone metastases, SRE outcomes, disease progression, and prognosis in cancer patients [19–22]. High levels of bone markers predict bone-related complications or SRE among cancer patients with bone metastases [23, 24]. In addition to increased risk of SRE occurrence, high bone ALP level before treatment is indicative of the progression of bone lesions and mortality [19]. Levels of total and bone ALP are significant predictors of prostate cancer-related death [21], and high bone ALP level is associated with shorter overall survival [25, 26]. In a retrospective analysis of the TAX327 study that included data from men with CRPC, bone metastases, and high baseline total ALP level who were receiving docetaxel or mitoxantrone, normalization of ALP level by day 90 predicted better survival while an increase in ALP level by day 90 predicted poor survival, both factors being independent of PSA decline [27]. Although the clinical significance of these bone markers is not well established, they do respond promptly and profoundly to bone-modulating agents (BMAs) and antineoplastic therapy, and this response appears to be associated with a favorable clinical outcome in patients with bone metastases [28].

From the biomarker analysis results in this study, total ALP levels in blood decreased by approximately 30% after the administration of radium-223 dichloride in all the cohorts at week 12, with approximately 55% of patients having a ≥30% reduction in total ALP. These results are comparable to those of the ALSYMPCA study: the mean percentage change in total ALP level from baseline at week 12 and EOT was −32.2 and −30.0%, respectively, and the ≥30% reductions of total ALP at week 12 and EOT were 46.9 and 60.1%, respectively [10]. No data on bone ALP are available in the ALSYMPCA study, but in this study the mean percentage change from baseline at week 12 and EOT was >45%, and a ≥30% reduction in bone ALP was seen in >81% of patients at week 12 and EOT. Since bone ALP is a specific marker for osteogenesis and is considered to be a reliable and established bone formation marker for prostate cancer with bone metastases [29], the decrease in the level of bone ALP of up to 50% during the treatment and high response rates are indicative of the anti-cancer activity of radium-223 dichloride against bone metastatic lesions, as well as the clinical benefit in this population.

Compared with markers of bone formation (total ALP, bone-specific ALP, P1NP), which were clearly decreased at 12 weeks after radium-223 dichloride administration (by ≥30%), the bone resorption markers CTX-1 and ICTP decreased to a lesser degree (by –20.8) or increased (by 14.3%), respectively. The lesser responsiveness of bone resorption markers is likely due to the use of BMAs, including denosumab and/or zoledronic acid, both prior to and during the study. BMAs inhibit bone resorption [30–33], and in cohort 1 and the expansion cohort, 12 of the 16 (75%) patients were pre-treated with BMAs before starting radium-223 dichloride therapy (data not shown). Pre-clinical studies have shown that bone-seeking α-emitters accumulate in the osteoblastic bone matrix [34]; therefore, the radium-223 dichloride-induced anti-tumor effects are expected to be concentrated in these lesions.

The strengths of this study include the rigorous methodology (inclusion and exclusion criteria, as well as the consistency achieved via the standard protocols defined in the internal manuals). This study, despite its small sample size, does confirm the results observed in ALSYMPCA, which was a large and controlled study [10].

Based on the results of the safety and efficacy analyses presented here, together with the report that there were no differences in the pharmacokinetics or the absorbed radiation dose in organs and tissues between Japanese and non-Japanese patients with CRPC and bone metastases receiving a single dose of radium-223 dichloride [35], the rational next step is to proceed to a Japanese phase II study for further efficacy evaluation. While the present study illustrates that treatment with radium-223 dichloride in Japanese patients decreased ALP, previous clinical trials in Caucasian patients have demonstrated that treatment of patients with CRPC and bone metastases with radium-223 dichloride confers a significant survival advantage, prolongs the time to SSEs and reduces the risk pf SSEs [10–12, 36]. Thus, further studies in Japanese patients should examine measures of survival and quality of life.

## Footnotes

1 The Japanese Society of Nuclear Medicine (NA) manual for proper use in clinical trials relating to radionuclide therapy with radium-223 dichloride (Ra-223) injection.

2 The Japanese Society of Nuclear Medicine (NA) Addendum to the manual for proper use in clinical trials relating to radionuclide therapy with Ra-223 dichloride (Ra-223) injection: methods of calculating radiation shielding and levels of radioactivity in the atmosphere, exhaust air and exhaust fluid.
